# How to exclude pulmonary embolism in patients hospitalized with COVID-19: a comparison of predictive scores

**DOI:** 10.1186/s12959-023-00492-5

**Published:** 2023-05-02

**Authors:** Jakob Vielhauer, Christopher Benesch, Anna Pernpruner, Anna-Lena Johlke, Johannes Christian Hellmuth, Maximilian Muenchhoff, Clemens Scherer, Nicola Fink, Bastian Sabel, Christian Schulz, Julia Mayerle, Ujjwal Mukund Mahajan, Hans Christian Stubbe

**Affiliations:** 1 Department of Medicine II, University Hospital LMU Munich, Munich, Germany; 2 German Center for Infection Research, Partner Site Munich, Munich, Germany; 3 Department of Medicine III, University Hospital LMU Munich, Munich, Germany; 4 COVID-19 Registry of the LMU Munich (CORKUM), University Hospital, LMU Munich, Munich, Germany; 5 Max von Pettenkofer Institute and Gene Center, National Reference Center for Retroviruses, Ludwig Maximilian University (LMU) of Munich, Virology, Munich, Germany; 6 Department of Medicine I, University Hospital LMU Munich, Munich, Germany; 7 Department of Radiology, Hospital of the LMU Munich, Munich, Germany

**Keywords:** Pulmonary embolism, COVID-19, Computed tomography pulmonary angiography, ventilation/perfusion scintigraphy, d-dimer

## Abstract

**Background:**

Pulmonary embolism (PE) is an important complication of Coronavirus disease 2019 (COVID-19). COVID-19 is associated with respiratory impairment and a pro-coagulative state, rendering PE more likely and difficult to recognize. Several decision algorithms relying on clinical features and D-dimer have been established. High prevalence of PE and elevated Ddimer in patients with COVID-19 might impair the performance of common decision algorithms. Here, we aimed to validate and compare five common decision algorithms implementing age adjusted Ddimer, the GENEVA, and Wells scores as well as the PEGeD- and YEARS-algorithms in patients hospitalized with COVID-19.

**Methods:**

In this single center study, we included patients who were admitted to our tertiary care hospital in the COVID-19 Registry of the LMU Munich. We retrospectively selected patients who received a computed tomography pulmonary angiogram (CTPA) or pulmonary ventilation/perfusion scintigraphy (V/Q) for suspected PE. The performances of five commonly used diagnostic algorithms (age-adjusted D-dimer, GENEVA score, PEGeD-algorithm, Wells score, and YEARS-algorithm) were compared.

**Results:**

We identified 413 patients with suspected PE who received a CTPA or V/Q confirming 62 PEs (15%). Among them, 358 patients with 48 PEs (13%) could be evaluated for performance of all algorithms. Patients with PE were older and their overall outcome was worse compared to patients without PE. Of the above five diagnostic algorithms, the PEGeD- and YEARS-algorithms performed best, reducing diagnostic imaging by 14% and 15% respectively with a sensitivity of 95.7% and 95.6%. The GENEVA score was able to reduce CTPA or V/Q by 32.2% but suffered from a low sensitivity (78.6%). Age-adjusted D-dimer and Wells score could not significantly reduce diagnostic imaging.

**Conclusion:**

The PEGeD- and YEARS-algorithms outperformed other tested decision algorithms and worked well in patients admitted with COVID-19. These findings need independent validation in a prospective study.

**Supplementary Information:**

The online version contains supplementary material available at 10.1186/s12959-023-00492-5.

## Introduction

Pulmonary embolism (PE) is an important complication of Coronavirus disease 2019 (COVID-19) [1]. Typical risk factors are malignancy, immobility, deep venous thrombosis, history of PE, obesity, pregnancy or a known coagulopathy [2]. COVID-19 is associated with a pro-coagulative state elevating the risk for PE and other thromboembolic events in patients with COVID-19. However, the diagnosis of PE is hampered as clinical symptoms of COVID-19 and PE are overlapping [1].

The gold standard for the diagnosis of PE is computed tomography pulmonary angiography (CTPA) and pulmonary ventilation/perfusion scintigraphy (V/Q) [3]. Other than V/Q, CTPA is widely accessible. Both methods have a high sensitivity and specificity regarding embolism detection [4]. In clinical routine, their usage is guided by decision algorithms based on D-dimer and clinical parameters if PE is suspected. Several decision algorithms have been established for managing suspected PE. Among the most widely used are the age-adjusted D-dimer (AAD), the revised GENEVA score (GENEVA), the Wells score with age adjusted D-dimer (WELLS), the YEARS-algorithm (YEARS) and the PEGeD-algorithm (PEGED) [5–9]. All these decision algorithms focus on high sensitivity to avoid missing the crucial diagnosis of PE. In lack of better biomarkers and unspecific clinical parameters, their specificity and positive predictive values are low. Consequently, a positive score requires confirmation by CTPA or V/Q while a negative score usually does not. The desired outcome of these algorithms is to reduce unnecessary CTPAs and V/Qs which are associated with radiation exposure, side-effects of contrast agents and expenditure of limited medical resources.

The clinical pictures of PE and COVID-19 are overlapping: symptoms like tachycardia, tachypnea, hypoxia or dyspnea are common features of both entities adding to the difficulty of identifying PE. This situation is further complicated by the association of COVID-19 with elevated levels of D-dimers, the key biomarker for PE [10].

Previous studies have validated the above scores in the pre-COVID-19 era, and few studies investigated their use in the emergency department setting and COVID-19 [11]. Generally, hospitalization is associated with an elevated risk of thromboembolic events. Hospitalization because of COVID-19 usually occurs in complicated or more severe disease stages which in turn is associated with higher levels of systematic inflammation [3]. Therefore, the accuracy of decision algorithms relying on clinical parameters and D-dimer might be severely impaired in this setting.

In this study, we aimed to validate and compare the five above mentioned decision algorithms for suspected PE in patients hospitalized with COVID-19.

## Methods

### Study population

This single-center study was conducted at our tertiary care hospital (LMU Klinikum of the Ludwig Maximilian University of Munich). We included patients who were hospitalized with COVID-19 in our prospective COVID-19 Registry of the LMU Munich (CORKUM) after informed consent was obtained. In the CORKUM cohort, we retrospectively identified all patients who received a CTPA or V/Q between 2/2020 and 6/2021 for suspicion of PE. We excluded patients with inconclusive CTPA or V/Q results or lacking clinical data. The ethics committee of the Medical Faculty of the Ludwig Maximilian University of Munich reviewed and approved the study protocols of CORKUM and this analysis.

### Imaging studies

CTPA was carried out using dual-source CT scanners after injection of a bolus of iodine-based contrast agent. The CTPA scans were interpreted by the attending radiologist and validated by a senior radiologist. Before V/Q, the patients received an x-ray image of the chest. Then, they inhaled a gaseous radionuclide and were injected radioactive technetium macro aggregated albumin. The images were acquired using a gamma camera. The scan was evaluated by the attending nuclear radiologists and validated by a senior nuclear radiologist.

### Data collection

Demographic and clinical data as well as CTPA and V/Q results were collected by investigators using the electronic data capture (EDC) software LCARS-C (LMU Klinikum, Germany). During data collection, the investigators were blinded towards decision rule results. The imaging study reports were documented in the EDC system only after complete documentation of demographic and clinical data.

**Fig. 1:**
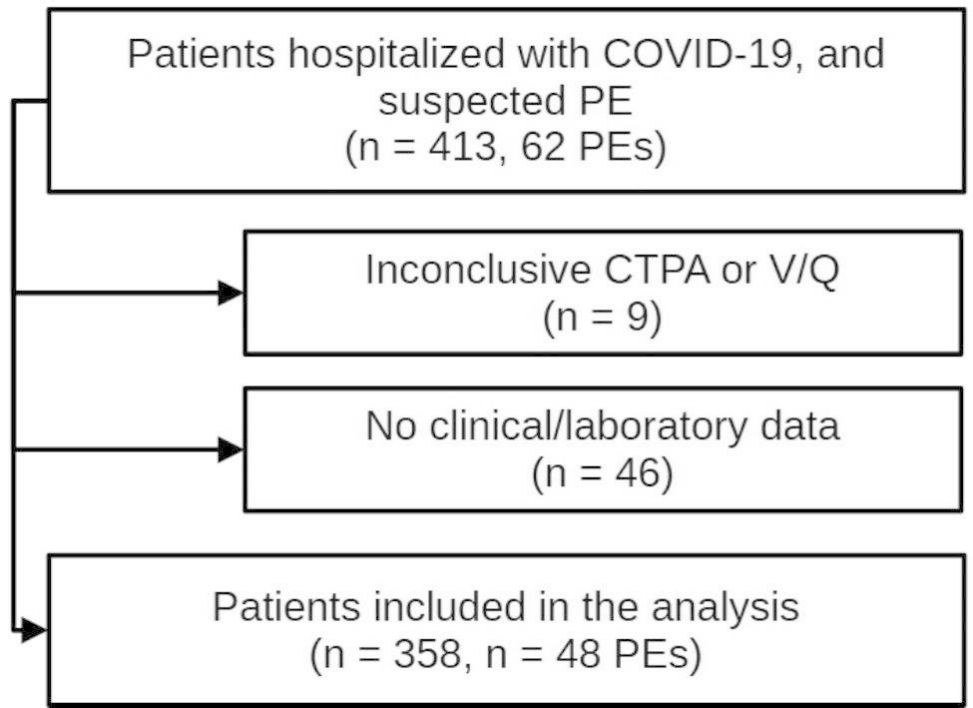
Flow-chart of patient selection for the analysis

### Decision algorithms

Clinical and laboratory data within 24 h before CTPA or V/Q scans were obtained. If multiple records were available, the timepoint raising the suspicion for PE was selected. After completion of data collection, the results of the decision algorithms were computed using an automated R script (R 4.2.1). Decision rule results were computed for AAD, GENEVA, PEGED, WELLS, and YEARS (Supplementary Table 1).

### Statistical analysis

Numeric variables are represented as medians with interquartile range (IQR). To test for statistically significant differences, we used a two-sided Kruskal-Wallis test. To test for correlations, we used the Spearman’s test together with the algorithm AS 89 to compute the respective p-values. Categorical variables are displayed as counts and frequencies. To test for statistically significant differences in count data, we used the Pearson’s Chi-squared test. Diagnostic test performance was assessed by calculating sensitivity, specificity, positive predictive values (PPV), negative predictive values (NPV), accuracies, and area under the curve (AUC) from the respective receiver operating characteristic curves (ROC) [12, 13]. Values are expressed together with their 95% confidence intervals (CI). We used the McNemar’s Chi-squared test with continuity correction to test for statistically significant differences in sensitivity and specificity between diagnostic tests. P-Values were corrected for multiple comparisons if applicable using the Benjamini & Hochberg method. P-values < 0.05 were considered significant. All statistical analyses and data visualization were carried out with R 4.2.1.

**Table 1:** **Baseline characteristica of patients included comparing pulmonary embolism (PE) and non-PE group.** A p-value < 0.05 was considered significant. Values are given as n (%) or median [IQR]. DVT (deep vein thrombosis), CRP (C-reactive protein), BMI (body mass index), IQR (inter quartile range).

Chracteristic	Subcategory	no PE	PE	p-value
**All**		N = 310	N = 48	
**Age**		65.1 [53.4;76.8]	73.2 [56.7;80.1]	0.054
**Sex**:				
	male	200 (64.5%)	32 (66.7%)	0.898
	female	110 (35.5%)	16 (33.3%)	
**Smoker**:				0.433
	former	27 (8.71%)	5 (10.4%)	
	no	251 (81.0%)	35 (72.9%)	
	unknown	18 (5.81%)	4 (8.33%)	
	yes	14 (4.52%)	4 (8.33%)	
**BMI**		34.8 [29.1;40.0]	40.7 [27.1;42.6]	0.500
**Reason for hospitalisation**				0.270
	COVID-19	279 (90.0%)	40 (83.3%)	
	other reason	24 (7.74%)	6 (12.5%)	
	unknown	7 (2.26%)	2 (4.17%)	
**Previous dvt or PE**		11 (3.55%)	3 (6.25%)	0.414
**Ongoing dvt**		0 (0.00%)	1 (2.08%)	0.134
**Arterial hypertension**		161 (51.9%)	26 (54.2%)	0.894
**Hematologic malignancy**		11 (3.55%)	0 (0.00%)	0.372
**Solid tumor**				0.193
	metastatic	10 (3.23%)	0 (0.00%)	
	no	278 (89.7%)	42 (87.5%)	
	unknown	17 (5.48%)	6 (12.5%)	
	yes	5 (1.61%)	0 (0.00%)	
**Symptoms/signs of dvt**		13 (4.19%)	6 (12.5%)	0.029
**Dyspnea**		152 (49.0%)	25 (52.1%)	0.812
**Chestpain**		43 (13.9%)	6 (12.5%)	0.975
**Abnormal fatigue**		102 (32.9%)	14 (29.2%)	0.727
**Fever**		132 (42.6%)	16 (33.3%)	0.292
**Cough**		124 (40.0%)	20 (41.7%)	0.951
**Dizziness**		22 (7.10%)	4 (8.33%)	0.764
**Hemoptysis**		7 (2.26%)	1 (2.08%)	1,000
**Oxygen saturation**		95.0 [92.0;96.0]	94.0 [92.5;96.0]	0.301
**Blood pressure systolic**		136 [121;150]	140 [126;156]	0.410
**Blood pressure diastolic**		79.0 [70.0;86.0]	80.0 [74.0;90.5]	0.189
**Pulse frequency**		90.0 [77.0;98.0]	90.5 [82.0;99.0]	0.686
**Glasgow Coma Scale**		15.0 [15.0;15.0]	15.0 [15.0;15.0]	0.669
**Laboratory measurements**				
	D-Dimer maximum value (µg/ml)	1.40 [1.00;2.70]	3.90 [1.60;11.4]	< 0.001
	CRP maximum value (mg/dl)	6.85 [3.42;12.4]	10.2 [5.50;14.6]	0.016
	Interleukin 6 maximum value (pg/ml)	60.4 [31.2;125]	93.7 [46.1;189]	0.013
	Hematocrit maximum value (l/l)	0.38 [0.34;0.42]	0.39 [0.36;0.43]	0.269
	Hemoglobin maximum value (g/dl)	13.1 [11.8;14.6]	13.4 [12.3;15.0]	0.213
	Glucose maximum value (mg/dl)	124 [108;152]	127 [105;148]	0.996
	Creatinine maximum value (Jaffé; mg/dl)	1.00 [0.80;1.30]	1.00 [0.80;1.30]	0.615
	Gamma-glutamyltransferase maximum value (U/l)	48.5 [29.0;91.0]	57.0 [36.8;140]	0.320
	Alanine transferase (ALT/GOT) maximum value (U/l)	46.0 [33.0;66.0]	49.0 [36.8;72.2]	0.289
	Aspartate aminotransferase (AST/GPT) maximum value (U/l)	36.0 [23.0;56.0]	44.0 [29.0;65.0]	0.138
	Creatine kinase maximum value (U/l)	116 [58.5;295]	118 [52.5;315]	0.994
**Admission worst COVID Stage (Leoss)**				0.147
	Complicated	100 (32.3%)	22 (45.8%)	
	Critical	23 (7.42%)	5 (10.4%)	
	Uncomplicated	178 (57.4%)	21 (43.8%)	
	Unknown	9 (2.90%)	0 (0.00%)	
**Outcome worst COVID stage (Leoss)**				0.362
	Complicated	128 (41.3%)	21 (43.8%)	
	Critical	72 (23.2%)	13 (27.1%)	
	Death	36 (11.6%)	8 (16.7%)	
	Uncomplicated	67 (21.6%)	5 (10.4%)	
	Unknown	7 (2.26%)	1 (2.08%)	
**Outcome discharge**				0.087
	Death	37 (11.9%)	8 (16.7%)	
	Discharge home	209 (67.4%)	24 (50.0%)	
	Discharge to care facility	50 (16.1%)	13 (27.1%)	
	Discharge to other hospital	9 (2.90%)	1 (2.08%)	
	Unknown	5 (1.61%)	2 (4.17%)	

**Fig. 2:**
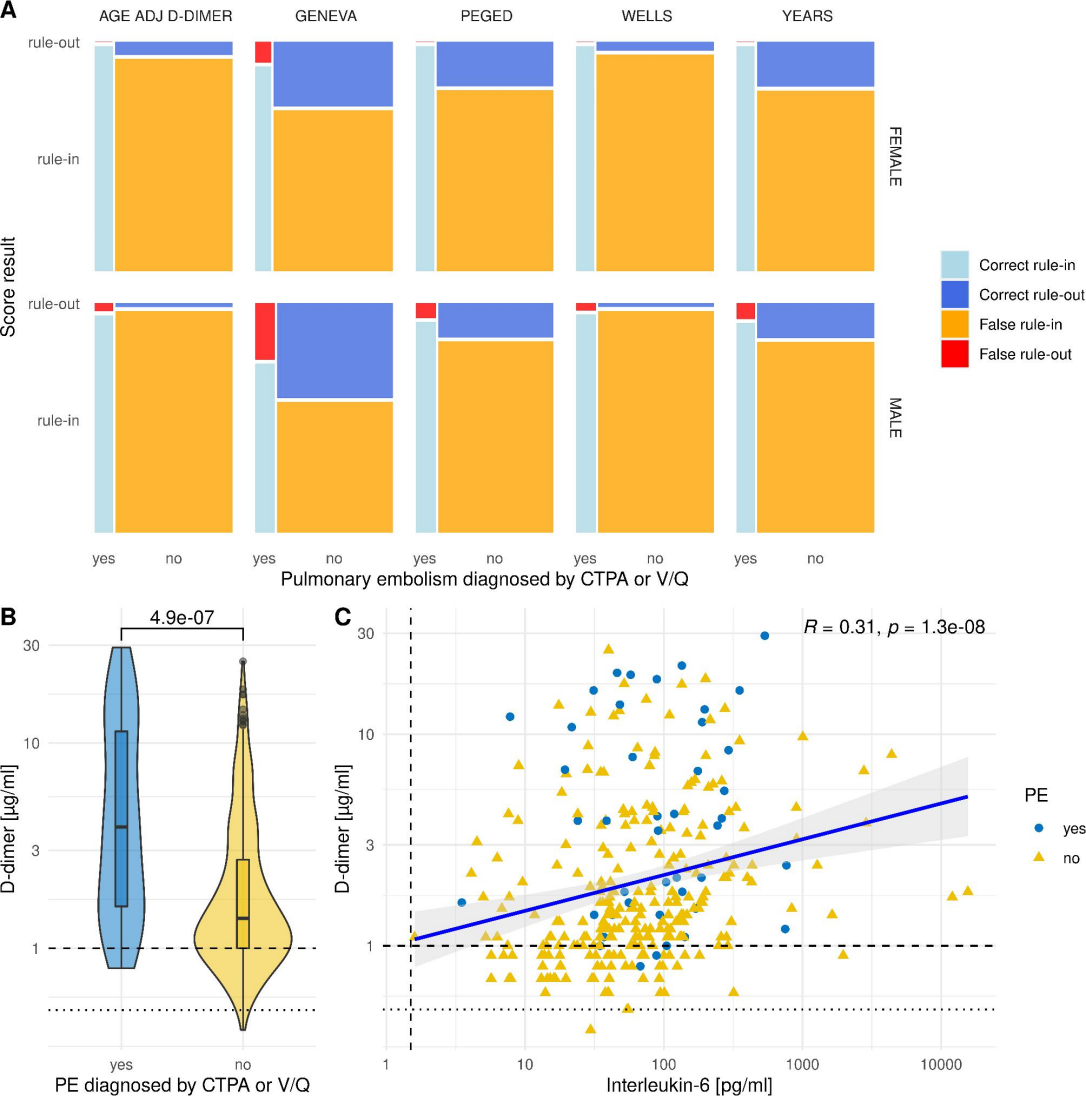
**Comparison of decision algorithms for PE in COVID-19.** (A) Mosaic diagram with each field’s size corresponding to the count of correct and false rule-in and -out (i.e., to the four fields of the respective contingency tables). False rule-out in red signifies missed PEs and the correct rule-out in dark blue corresponds to correctly avoided CTPA or V/Q. (B) Violin plot of D-dimer levels, comparing PE versus no PE. The p-value is calculated using a two-sided Mann-Whitney test. The dashed line corresponds to a Ddimer of 1 µg/ml; the dotted line to 0.5 µg/ml. (C) Spearman correlation of Ddimer and interleukin-6. The vertical dashed line corresponds to an interleukin-6 of 1.5 pg/ml; values < 1.5 pg/ml are considered normal. Note the log-transformed axes for d-dimer and interleukin-6 in (B) and (C)

**Table 2:** **Performance of different scores in predicting pulmonary embolism (PE)**. All values are given with 95% confidence intervals, except counts. These are shown as n (%). AAD (age-adjusted D-dimer), CTPA (computed tomography pulmonary angiogram)

	AAD	GENEVA	PEGED	WELLS	YEARS	YEARS + IL-6
**Sensitivity**	0.978 [0.935,1.00]	0.800	0.957	0.979	0.956	0.911
**Specificity**	0.031 [0.011,0.050]	0.371	0.167	0.024	0.169	0.192
**Positive predictive value**	0.148 [0.110,0.185]	0.180	0.165	0.147	0.165	0.163
**Negative predictive value**	0.888 [0.693,1.00]	0.915	0.958	0.868	0.957	0.926
**Correct rule-out (CTPA/scintigraphy avoided)**	9.00 (2,65%)	85.00 (32.20%)	47.00 (14.33%)	7.00 (2.03%)	50.00 (14.71%)	55.00 (16.57%)
**False rule-out (PEs missed) n (%)**	1.00 (0.29%)	7.00 (2.65%)	2.00 (0.61%)	1.00 (0.29%)	2.00 (0.59%)	4.00 (1.2%)
**Accuracy**	0.1559 [0.119,0.1989]	0.428 [0.3676,0.4901]	0.2805 [0.2325,0.3325]	0.1565 [0.1198,0.1992]	0.2735 [0.2268,0.3242]	0.2892 [0.241,.03412]
**Area under the curve (AUC)**	0.723 [0.634,0.811]	0.715 [0.614, 0.816]	0.705 [0.604, 0.807]	0.711 [0.609, 0.812]	0.741 [0.654,0.827]	

## Results

We identified a total of 413 inpatients with COVID-19 who received CTPA or V/Q for suspected PE of whom 62 (15%) were diagnosed with PE. Among them, 358 patients with 48 PEs (13%) had conclusive imaging results and complete records of clinical and laboratory parameters, allowing for the computation of the five included decision algorithms (Fig. 1).

**Fig. 3:**
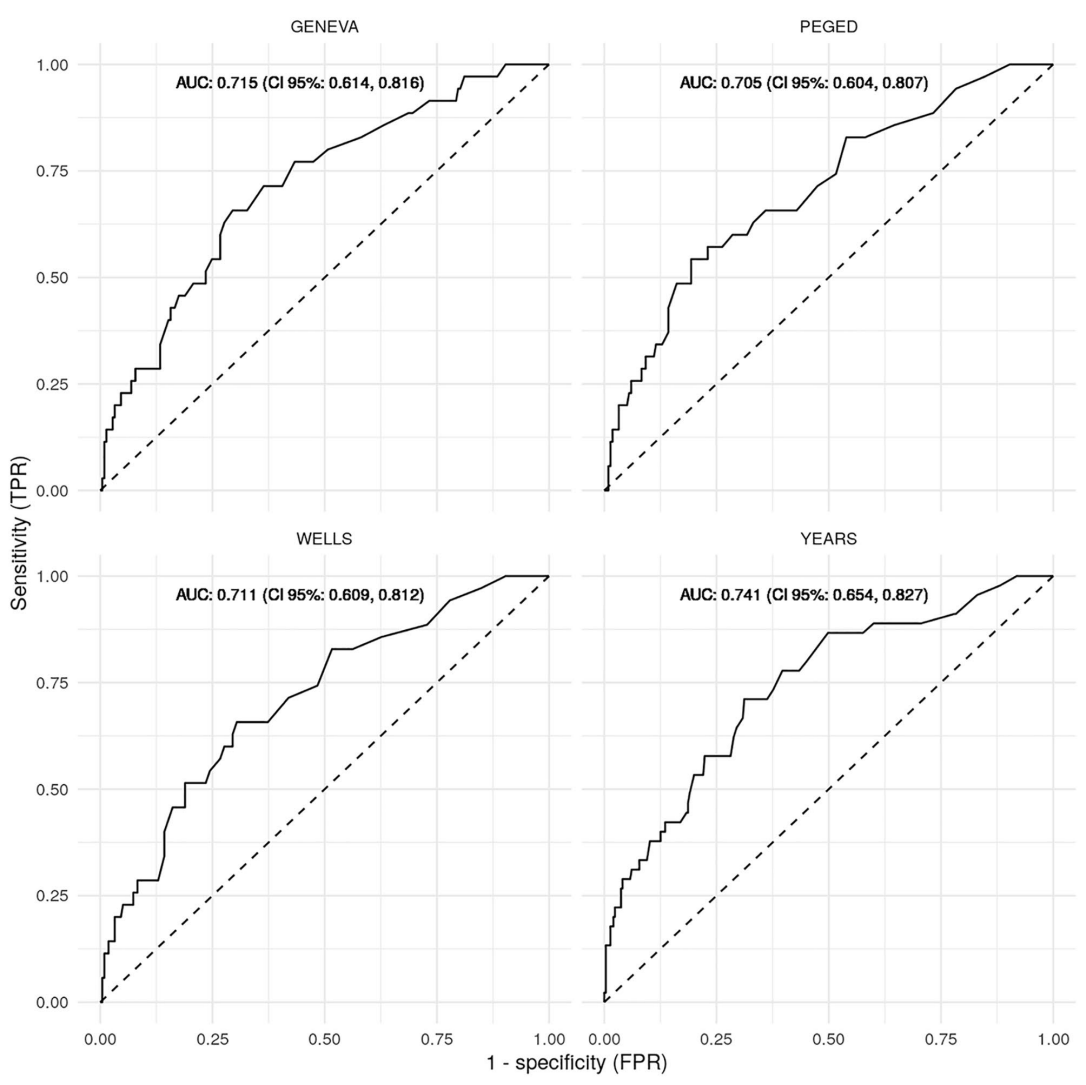
**ROC curves and AUCs.** Four ROC curves were plotted for GENEVA, PEGED, WELLS and YEARS. The dashed lines represent the chance lines, where the AUC would be 0.5, corresponding to a random prediction. The curves were computed adjusting the D-dimer levels by each rule to create meaningful ROC curves. AUCs are given with 95% CI.

The median age of patients with PE was higher compared to non-PE patients, but not statistically significant (73.2 [56.7;80.1] and 65.1 [53.4;76.8], respectively; p = 0.054). Frequencies of sex, smoking status or body mass index did not show any significant differences (Table 1). Likewise, frequencies of significant comorbidities were similar in both groups. Notably, the only clinical signs and symptoms, differing significantly (p = 0.029) between the two groups were those of deep vein thrombosis. They were more frequent in the PE group with a frequency of 12.5% compared to 4.2% in the non-PE group. Other hall-mark symptoms of PE, such as tachycardia, dyspnea, reduced peripheral oxygen saturation or chest pain occurred at similar rates without any significant differences, highlighting the difficulty of identifying PE in patients hospitalized with COVID-19. Laboratory results showed a highly significant (p < 0.001) increase in D-dimer as expected (in PE-patients 3.90 µg/ml [1.60;11.4] compared to 1.40 µg/ml [1.00;2.70] in non-PE-patients, respectively). Similarly, we observed a marked increase of inflammatory markers in PE patients compared to non-PE such as a median interleukin-6 (IL6) of 93.7 pg/ml [46.1;189] versus 60.4 pg/ml [31.2;125] (p = 0.013) or a median C-reactive protein (CRP) of 10.2 mg/dl [5.50;14.6] compared to 6.85 mg/dl [3.42;12.4] (p = 0.016) indicating the interaction between coagulation and inflammation (Fig. 2B-C).

Altogether, 89% of the included patients were hospitalized because of COVID-19 without significant differences between PE and non-PE patients. No differences were detected for COVID-19 severity on admission, or during the hospital stay (Table 1 and Supplementary Table 2). Regarding the overall outcome at discharge, we observed more severe disease courses in PE patients compared to non-PE patients such as death (17% versus 12%) or discharge to care facility (27% versus 16%). However, this was not statistically significant (p = 0.087). Overall, 13% of patients were diagnosed with PE using CTPA (99.7%) or V/Q (0.3%). The overall PE prevalence in our population was close to the expected overall prevalence of PE in patients hospitalized with COVID-19 [14]. Most pulmonary embolisms detected in this study were segmental (54%), followed by central (21%), and sub-segmental embolisms (6%).

We computed the results of five common clinical decision algorithms from the available clinical and laboratory data: AAD, WELLS, GENEVA, PEGED and YEARS. AAD and the WELLS had very low overall accuracies at 0.156 (95% CI: 0.119, 0.199) and 0.156 (95% CI: 0.120, 0.199). The accuracies of YEARS and PEGED were significantly higher with 0.273 (95% CI: 0.227, 0.324) and 0.280 (95% CI: 0.232, 0.332), respectively. The highest accuracy was observed for GENEVA at 0.428 (CI 95%: 0.368, 0.490). Since the D-dimer was above 0.5 µg/ml in most patients, the performances of AAD and WELLS were skewed towards a high sensitivity of 0.978 (95% CI: 0.935, 1.000) and 0.979 (95% CI: 0.939, 1.000) at the cost of a very low specificity 0.031 (95% CI: 0.011, 0.050) and 0.024 (95% CI: 0.006, 0.041). In consequence, both ruled-out PEs avoiding CTPA or V/Q in only 2.6% and 2.0% of the cases while false rule-out occurred in 2.2% and 2.1%. The specificity of GENEVA was significantly better compared to the former two decision algorithms with 0.371 (95% CI: 0.309, 0.434) at the cost of a significantly reduced sensitivity at 0.800 (95% CI: 0.667, 0.933). As a result, GENEVA could correctly rule out more than the other decision algorithms avoiding 32.3% imaging studies but missing 20% of the actual PEs. The specificity of PEGED and YEARS was at 0.167 (CI 95%: 0.124, 0.211) and 0.169 (CI 95%: 0.127, 0.212) significantly better comparing to AAD and WELLS, but not as good as GENEVA. Both decision algorithms showed high sensitivity with 0.957 (CI 95%: 0.900, 1.000) and 0.956 (CI 95%: 0.985, 1.000). PEGED and YEARS correctly ruled out 14.3% and 14.7% while missing only 4.3% and 4.4% of the actual PEs. Given the overall prevalence of 15% for PEs in patients hospitalized with COVID-19, negative predictive values (NPV) were quite high for the five decision algorithms with highest values at 0.958 (CI 95% 0.902, 1.000) for PEGED and 0.957 (CI 95% 0.901, 1.000) for YEARS, followed by 0.915 (CI 95% 0.858, 0.972) for GENEVA. AAD and WELLS had lower values with NPVs at 0.888 (CI 95%: 0.693, 1.000), and 0.868 (CI 95%: 0.633, 1.000), respectively (Fig. 2A; Table 2).

In order to compute meaningful ROC curves and AUCs, we used D-dimer levels as the dependent variable which we modified by the results of each decision rule. Here, the AUCs ranged between 0.711 (CI 95%: 0.614, 0.816) for GENEVA and 0.741 (CI 95%: 0.654, 0.827) for YEARS. Overall, the differences in AUC were marginal and statistically non-significant. This indicates that the above differences in performance were mostly due to optimized cutoffs (Fig. 3).

In almost all patients, D-dimers and inflammation markers were elevated, which was significantly more pronounced in the PE-group. As expected from a clinical point of view, we observed a highly significant correlation of D-dimer with CRP and IL6 (R 0.29, p < 0.0001 and R 0.31, p < 0.0001; Fig. 2C). Since the YEARS algorithm uses only two fixed D-dimer cutoffs and performed very well, we tested the YEARS score adjusting D-dimer cutoff for IL6. We adjusted the D-dimer cutoffs by an increased 0.1 µg/dl within the YEARS-algorithm if patients had an IL-6 of 10 pg/ml or higher to either 0.6 µg/ml, or to 1.1 µg/ml if all YEARS items were negative. The IL-6 adjusted YEARS algorithm had an increased accuracy of 0.289 (95% CI: 0.241, 0.341), but its sensitivity decreased to 0.625 (95% CI: 0.290, 0.960).

## Discussion

Early during the pandemic, high rates of thromboembolic events such as PE were noted in COVID-19 patients [14]. The disease is associated with activated inflammation which drives a pro-coagulative state. Patients hospitalized with COVID-19 are usually more severely affected by the disease and exhibit an elevated risk for PE [15, 16]. Unfortunately, the identification of PE is more difficult in patients with COVID-19 because of overlapping symptoms, an elevated risk of PE and increased D-dimer values.

Previous studies validated AAD, GENEVA, PEGED, WELLS, or YEARS in the context of COVID-19 but focused on the emergency department [11]. Data validating the above algorithms for an in-patient cohort is lacking. Given the special situation of hospitalized patients with COVID-19, validation of diagnostic algorithms is urgently required.

In this retrospective cohort study, we tested five clinical decision algorithms for their ability to guide the diagnostic approach of suspected PE in patients hospitalized with COVID-19. We found that GENEVA was most accurate but suffered from limited sensitivity and a poor NPV. PEGED and YEARS performed best regarding NPV while not compromising on safety. AAD and WELLS showed very high sensitivity but did not add diagnostic information in ruling out PE, since they classified nearly all patients into the PE group. Compared to previous studies in non-COVID-settings, PEGED and YEARS performed comparable regarding accuracy, specificity and sensitivity, while the other three decision algorithms performed worse [8, 9, 17, 18]. All algorithms showed similar AUCs indicating that their main clinical feature was D-dimer with different finetuning towards cut-offs.

A key problem in ruling out PE is the elevated D-dimer often observed in COVID-19, an effect which aggravates with increasing severity and inflammation [10]. In our study, most patients had elevated Ddimers. PEGED and YEARS, the two best performing decision algorithms, allowed for an increased D-dimer cutoff if clinical markers signaled a low pretest probability. The two least performant performing decision algorithms, AAD and WELLS, used a D-dimer cutoff of 0.5 µg/ml or a conservative age-adjustment, which was not sufficient to account for the increased D-dimer levels in patients suffering from COVID-19. This led to a very low specificity, rendering the two decision algorithms nearly irrelevant in our cohort. While GENEVA performed substantially better, its sensitivity was worst. Possibly, this is due to the fact, that GENEVA does not incorporate the gestalt item “is PE the most probable diagnosis?”.

The results of WELLS and PEGED differed significantly, even though both make use of the Wells items. PEGED is based on the three-tier Wells score, grouping patients into low, intermediate, and high clinical pretest probability (C-PTP). In their original study, Wells and colleagues used a two-tier model (high and low C-PTP) and measured D-dimer in all patients with suspected PE. If the C-PTP was low and the D-dimer was < 0.5 µg/dl (i.e. negative), PE was considered as excluded [18]. PEGED, on the other hand, accepts a D-dimer cutoff of < 1.0 µg/dl for patients with low C-PTP, a D-dimer cutoff of 0.5 µg/dl for intermediate C-PTP, while a high C-PTP prompts further diagnostic evaluation (e.g. CPTPA or V/Q). Apparently, the key difference between the two approaches is, that the PEGeD-algorithm accepts a D-dimer cutoff of < 1.0 µg/dl for a low C-PTP. Similarly, the YEARS algorithm accepts a D-dimer cutoff of < 1.0 µg/dl, when all YEARS items are negative. This explains, why the PEGED results differ strongly from the Wells results, while resembling the results of YEARS.

To optimize the YEARS algorithm, we adjusted the D-dimer cutoffs depending on IL-6-levels. While a slightly increased cutoff for patients with an IL-6 > 10 pg/ml yielded better accuracy, sensitivity suffered, and more missed PEs would have been the consequence. It seems that using inflammation adjusted cutoffs is a double-edged sword. An increase in cutoffs might account for the increased D-dimer levels but mitigate sensitivity in a situation with increased risk of PE.

This study has important limitations. Patients for this analysis were selected retrospectively from the prospective CORKUM registry, elevating the risk for bias compared to a purely prospective study. This study was conducted at a single tertiary care center in Munich, Germany. While the study patients comprised a range of features, patient characteristics might differ significantly in other settings (e.g. primary care centers) or other geographic regions. Important strengths of this study are, that several scores were evaluated and that the study focused on hospitalized patients. Identification of PE is of particular interest in hospitalized patients, since the risk for PE is elevated in these patients, highlighting the importance of this study.

## Conclusion

This study showed, that PEGED and YEARS are reliable methods for guiding the diagnostic approach when PE is suspected in patients hospitalized with COVID-19. These findings add to the evidence supporting PEGED and YEARS as diagnostic tools in settings when PE is suspected.

## Electronic supplementary material

Below is the link to the electronic supplementary material.

Supplementary Material 1

## Data Availability

All clinical data will be made available upon reasonable request. The analysis scripts are included in the supporting information.

## List of Abbreviations

AAD: Age-adjusted D-Dimer
AUC: Area under the curve
CI: Confidence interval
COVID-19: Coronavirus disease 2019
C-PTP: Clinical pretest probability
CRP: C-reactive protein
CTPA: Computed tomography pulmonary angiogram
EDC: Electronic data capture
GENEVA: Revised GENEVA algorithm
IL6: Interleukin-6
IQR: interquartile range
NPV: Negative predictive value
PE: Pulmonary embolism
PEGED: PEGeD-algorithm
PPV: Positive predictive value
ROC: Receiver operating characteristic curves
RT-PCR: Reverse transcription polymerase chain reaction
V/Q: Ventilation/perfusion scintigraphy
WELLS: Well’s score with age adjusted D-Dimer
