# Supplementary material for: How to exclude pulmonary embolism in patients hospitalized with COVID-19: a comparison of predictive scores
Source: Thromb J. 2023 May 2;21:51. doi: 10.1186/s12959-023-00492-5 (PMC10153021; doi:10.1186/s12959-023-00492-5)
Supplement: Supplementary file 1 — Supplementary Material 1 [file 12959_2023_492_MOESM1_ESM.pdf]

How to exclude pulmonary embolism in patients hospitalized with COVID-19: a comparison of predictive scores

Supplementary tables

Supplementary Table 1. Decision rules and scores.

Score	Items (points if applicable)	Decision rule
**Age-adjusted D-Dimer**	None	• 50 years or younger: D-dimer cut-off: 0.5 µg/ml<br>• >50 years: D-dimer cut-off = 0.1 patient age µg/ml
**YEARS algorithm**	• Clinical signs of DVT<br>• Hemoptysis<br>• PE most likely	• 0 items and D-dimer < 1 µg/ml: PE excluded.<br>• ≥ 1 item and D-dimer < 0.5 µg/ml: PE excluded
**Wells Score**	• Clinical signs of DVT (3)<br>• PE most likely (3)<br>• Heartrate >100/min (1,5)<br>• Immobilization > 3d/ surgery <4 weeks (1,5)<br>• History of PE/DVT (1,5)<br>• Hemoptysis (1)<br>• Neoplasia (1)	• Sum ≤ 4 and age-adjusted D-dimer negative: PE excluded.<br>• Sum > 4: PE not excluded
**PEGeD algorithm**	• Wells items	• Sum ≤ 4 and D-dimer < 1 µg/ml: PE excluded.<br>• Sum > 4 and ≤ 6 and D-dimer < 0.5 µg/ml: PE excluded.<br>• Sum > 6: PE not excluded.
**Revised GENEVA Score**	• Age > 65 y (1)<br>• History of PE/DVT (3)<br>• Surgery/bone fracture <4 weeks (2)<br>• Neoplasia (2)<br>• Unilateral leg pain (3)<br>• Unilateral leg swelling/painful palpation (4)<br>• Hemoptysis (2)<br>• Heartrate 75-94/min (3); >94/min (4)	• Sum ≤ 3: PE excluded.<br>• Sum > 3 and ≤ 10 and age-adjusted D-Dimer negative: PE excluded.<br>• Sum > 10: PE not excluded.

DVT: deep vein thrombosis, PE: pulmonary embolism

Supplementary Table 2. Criteria for different disease stages, according to LEOSS (1).

**Uncomplicated Stage**	**Complicated Stage**	**Critical Stage**
Asymptomatic OR	Need for new - or meaningful increase in prior - oxygen supplementation	Need for catecholamines
Symptoms of upper respiratory tract infection	PaO2 at room air < 70mmHg/ SO2 at room air < 90%	Life-threatening cardiac arrythmia
Nausea, emesis, diarrhea	AST/ALT > 5x ULN	qSOFA >=2
Fever	New cardiac arrythmia	Acute renal failure in need for dialysis
	New pericardial effusion >1cm	Liver failure with Quick <50% or INR > 3.5
	New heart failure with pulmonary edema, congestive hepatopathy/peripheral edema	Need for unplanned mechanical ventilation (invasive or non-invasive)/ prolongation (>24h of planned mechanical ventilation)

ULN (upper level of normal), AST (aspartate transaminase), ALN (alanin transaminase).

1. Jakob CEM, Borgmann S, Duygu F, Behrends U, Hower M, Merle U, u. a. First results of the “Lean European Open Survey on SARS-CoV-2-Infected Patients (LEOSS)”. Infection [Internet]. 1. Oktober 2020 [zitiert 14. Oktober 2020]; Verfügbar unter: https://doi.org/10.1007/s15010-020-01499-0
